# Recognition software successfully aids the identification of individual small‐spotted catsharks *Scyliorhinus canicula* during their first year of life

**DOI:** 10.1111/jfb.14166

**Published:** 2019-11-06

**Authors:** Samantha A. Hook, Charlotte McMurray, Daniel M. Ripley, Natasha Allen, Timo Moritz, Bianka Grunow, Holly A. Shiels

**Affiliations:** ^1^ Division of Cardiovascular Sciences, Faculty of Biology, Medicine and Health University of Manchester, Core Technology Facility Manchester UK; ^2^ Biological Services Facility University of Manchester Manchester UK; ^3^ Deutsches Meeresmuseum Stralsund Germany; ^4^ Institut für Zoologie und Evolutionsforschung, Friedrich‐Schiller‐Universität Jena Jena Germany; ^5^ Leibniz‐Institute for Farm Animal Biology Dummerstorf Germany

**Keywords:** captivity, conservation, elasmobranchs, I^3^S, management, microsatellites

## Abstract

Eighteen captive small‐spotted catsharks *Scyliorhinus canicula* were successfully identified from hatching to 1 year of age using the free computer recognition software, I^3^S classic. The effect of increasing the time interval between recognition attempts on the accuracy of the software was investigated, revealing that recognition fiedelity decreases with increasing time intervals for younger (0 to 15 weeks), but not older (15 weeks onwards) sharks. Identification by I^3^S was validated using genetic analyses of seven microsatellite markers, revealing a 100% success rate. Thus, this non‐invasive recognition method can be used as an inexpensive and effective alternative to invasive tagging, improving animal welfare and complementing *ex‐situ* conservation methods.

## 1 INTRODUCTION

Identification of individual animals is often crucial in studies of wild and captive populations (Marshall & Pierce, 2012). Physical tags, such as T‐bar anchors and PIT tags are commonly used on fish. However, these methods of identification can be limited by tag loss, negative effects on growth, health and escape from predators, as well as injury or even death from tag application (Cailliet *et al*., 1992; Feldheim *et al*., 2002; French *et al*., 2015; Manire & Gruber, 1991). Furthermore, many physical tags are too large to use on young or small individuals. Animal biometrics offers a non‐invasive and economical alternative to invasive identification methods. Natural markings, scars and contours that are unique to individuals and that are maintained throughout their lives have been used for individual recognition across a variety of marine taxa including pinnipeds, cetaceans, sirenians and elasmobranchs (Gubili *et al*., 2009; MacLeod, 1998; Pawley *et al*., 2018; Wells, 2018). Such databases can grow large and performing the identifications manually can become time‐inefficient. Photo‐recognition is often used, therefore, alongside invasive and non‐invasive tagging methods as a tool to determine and track morphological changes, in addition to providing individual identification (ID; Chin *et al*., 2015). Recognition software, such as the freeware Interactive Individual Identification System Classic (I^3^S; http://www.reijns.com), can aid the process of manual identifications and possibly remove the need for invasive tagging. I^3^S has been successfully used for individual identification in the whale shark *Rhincodon typus* Smith 1828 (Graham & Roberts, 2007; Speed *et al*., 2007), the white shark *Carcharodon carcharias* (L. 1758) (Andreotti *et al*., 2016, 2018), the spotted eagle ray *Aetobatus narinari* (Euphrasen 1790) (González‐Ramos *et al*., 2017), the ragged‐tooth or grey nurse shark *Carcharias taurus* Rafinesque 1810 (Bansemer & Bennett, 2008; van Tienhoven *et al*., 2007) and the lesser‐spotted catshark Scyliorhinus canicula (L. 1758) (Navarro *et al*., 2018). I^3^S software produces recognition scores derived from the distances between pairs of spots that are chosen by the user (van Tienhoven *et al*., 2007). Using reference points, the distances between the spots are scaled to the size of the animal, so growth should not be detrimental to recognition. However, to our knowledge, biometrics and I^3^S software have not been used during early development in fish.

A growing number of studies are focussing on early life‐stages in fishes but their size and vulnerability render many standard tagging regimes inadequate. Here, the ability of I^3^S to recognise individual, juvenile small‐spotted catshark *Scyliorhinus canicula* (L. 1758) during their first year of life was investigated. We examined the efficacy of I^3^S in identifying individuals through time by comparing matches to those generated with microsatellite marker analysis. Finally, we suggest a photography regime that maximises the efficiency of I^3^S as a tool for laboratory use.

## 2 MATERIALS AND METHODS

All procedures complied with the ethical review board of the University of Manchester.

### 2.1 Study fish

The study population consisted of 18 *Scyliorhinus* (sex not determined) that arrived at The University of Manchester (UK) as embryos from the OZEANEUM, Stralsund, Germany (http://www.ozeaneum.de). In the laboratory, the egg cases were transferred into 45 l static seawater tanks that were maintained at 15°C and salinity 35, under a 12:12 h light: dark cycle, until hatching. After hatching, the sharks were held in three 400 l tanks under the same conditions as during embryogenesis.

### 2.2 Photography of the hatchlings

Photographs of the 18 individuals were taken once a week for 7 weeks after hatching. Additional photographs were taken at weeks 12, 14, 16, 30, 32 to 34, 38, 44 and 45. The age of the hatchlings when the first photograph was taken ranged from 0 to 2 weeks for 14 individuals; the remaining 4 individuals had their first photograph at 16 weeks post‐hatch; thus, these animals were 60 weeks of age at the end of the study.

Each week's photographs were stored in a separate database containing all the individuals for the given week. A Sony Cyber‐shot T300 camera (http://www.sony.jp) and a Moto G3 phone camera (Motorola; http://www.motorola.com) were used for the photography. Using a net, the sharks were transferred individually from their holding tanks to a small transparent test tank (*c*. 12 × 20 cm) containing enough water to submerge the specimen. The lid of the tank was removed and the dorsal side of the animal was photographed parallel to the camera lens to avoid distortion. Afterwards, individuals were returned to their original tank. By the end of the study period three individuals had died from unknown causes but the data provided by these individuals for the earlier time points were not removed from the databases.

### 2.3 Data input into I^3^S

**Figure 1:**
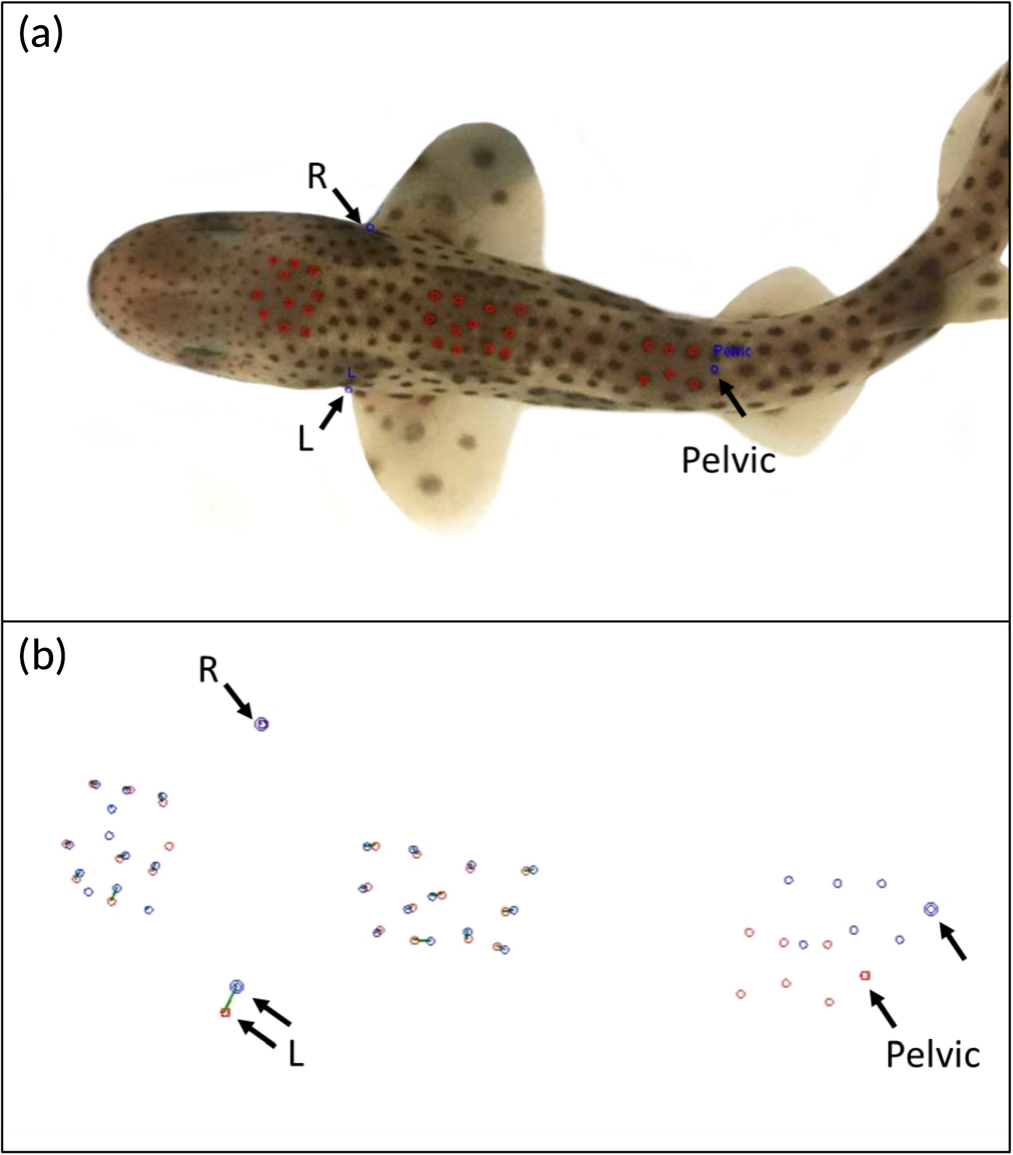
**(**a) Reference points (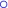, 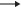) for input patterns for I^3^S: the corners of the right (R) and left (L) pectoral fins and the midpoint between the pelvic fins (Pelvic) of *Scyliorhinus canicula*. Up to 30 natural patterning spots on the hatchling are selected by the user (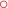). (b) Comparisons of two different individual *S. canicula* patterns in I^3^S. Individual 1 is the catshark from (a) with teh spot pattern from a different individual (not shown) overlaid. The same three reference regions were used for all sharks. Note the right (R) pelvic fin references points are overlaid. The software identifies where a marking is the same between individuals. The greater the number of overlaid marks or joined lines between marks, the closer the two patterns are to each other and thus the better (i.e., the lower) the recognition score

I^3^S requires manual input of an animal's patterns from photographs into databases. Three reference points were selected, which correct for discrepancies in angle and scale between two photos. The three reference points chosen for pattern input in this study were the anterior corners of the right and left pectoral fins where they meet the body and the central point between the anterior corners of the pelvic fins (Figure 1a). The individual's spots were then pinpointed by a single user throughout the study (Figure 1a), creating a two‐dimensional pattern that the software compares automatically to the rest of a given database by overlaying the 2D patterns (Figure 1b). With I^3^S, a maximum of 30 spots can be selected. As *S. cannicula* usually develop more than 30 spots, the most prominent spots on the dorsal side were pinpointed by the user.

### 2.4 Data output from I^3^S – recognition score

I^3^S software produces a recognition score derived from the distances between pairs of spots in the images being compared (van Tienhoven *et al*., 2007). Recognition scores demonstrate the closeness of a match between a given image and every other image in the given database. The lower the recognition score, the more similar the patterns are between images; a recognition score of 1 presents the user with the perfect match. I^3^S produces a ranked list of potential matches for the query image against all the images in the given database. The user is then required to visually interrogate the potential matches to determine the true match. In this respect I^3^S is a valuable aid for photographic identification but does not replace the need for visual checking by the user. Indeed, most studies employ visual confirmation of the computed matches (Andreotti *et al*., 2016, 2018; González‐Ramos *et al*., 2017; Speed *et al*., 2007; van Tienhoven *et al*., 2007). However, genetic validation of matches is less commonly used (Graham & Roberts, 2007) and never, to our knowledge, during early development.

To establish how age relates to changes in the patterning of individuals and thus how frequently photographs must be taken to track an individual over time, separate databases containing images of each animal in I^3^S were created for each week, producing a database time series. This time‐series of databases was used to compute recognition scores for each individual across increasing time intervals in order to determine how the time between photographs affects the performance of the software. The recognition scores produced by I^3^S that were associated with the correct match determined by eye were recorded.

### 2.5 Genetic analysis

Fin clips were taken from each individual at the beginning and end of the experimental period (listed as Cat01–Cat18 and HAM43–HAM57, respectively; Table 1). Three individuals died of natural causes during the experimental period (Cat11, Cat15 and Cat18), leaving 15 individuals genetically identified at the end of the study. The entire population of potential parents (four males and three females) from the captive source population housed at the OZEANEUM were also fin clipped and added to the sample set to allow us to account for siblingship, which affects the precision of genetic identification.

**Table 1:** The genetic matches between first and final set of samples for *Scyliorhinus canicula*

Initial ID	Final ID	Matching loci	Mismatching loci	pID	Photo‐ID match status
Cat01	HAM48	7	0	4.10E‐05	Exact match
Cat02	HAM46	7	0	1.64E‐04	Exact match
Cat03	HAM44	7	0	1.18E‐05	Exact match
Cat04	HAM51	7	0	1.83E‐04	Exact match
Cat05	HAM45	7	0	2.01E‐04	Exact match
Cat06	HAM43	7	0	1.07E‐04	Exact match
Cat07	HAM47	7	0	1.40E‐06	Exact match
Cat08	HAM57	7	0	4.22E‐05	Exact match
Cat09	HAM52	7	0	1.46E‐04	Exact match
Cat10	HAM56	7	0	3.23E‐06	Exact match
Cat12	HAM55	7	0	2.07E‐04	Exact match
Cat13	HAM54	7	0	6.04E‐04	Exact match
Cat14	HAM50	7	0	1.22E‐04	Exact match
Cat16	HAM53	7	0	6.87E‐05	Exact match
Cat17	HAM49	7	0	2.80E‐05	Exact match

Note: ID: individual identification; pID: probability of identity.

Genomic DNA from the fin clips was extracted using Bioline Islotte II blood and tissue kit (Diatek; http://www.diatek.ch.) Samples were amplified using seven microsatellite primers (*Scan‐02*, *Scan‐04*, *Scan‐09*, *Scan‐10*, *Scan‐12*, *Scan‐15*, *Scan‐16*) (Griffiths *et al*., 2011). The products were genotyped at the University of Manchester sequencing facility and scored using GeneMapper 4.1 (Applied Biosystems; http://www.appliedbiosystems.com). Alleles were validated using MicroChecker (van Oosterhout *et al*., 2004) and the genotypic fingerprints were run through the program CERVUS to determine probability of identity analysis (pID; Marshall *et al*., 1998, Kalinowski *et al*., 2007). Parentage analysis using the program CERVUS was conducted to determine the extent of siblingship of the offspring (*i.e*., full siblings, half siblings or unrelated).

## 3 RESULTS

### 3.1 The effect of photographic time interval on accuracy of photo recognition

The three reference points (Figure 1a) were held consistent between all input patterns to correct for size and angle. Two‐dimensional pattern comparisons of separate individuals can be visualised by I^3^S and are shown in (Figure 1b).

**Figure 2:**
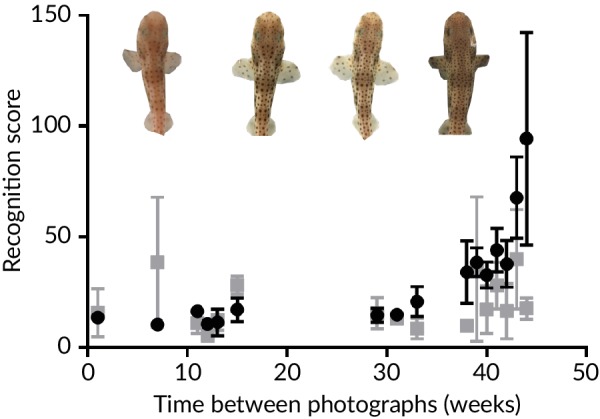
Comparison of mean (±SE) *Scyliorhinus canicula* spot‐pattern recognition score in I^3^S among older (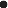) and younger (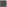) individuals. The photographs show the spot pattern in one shark at (left to right) 16, 29, 45 and 60 weeks of age. *n.b*., In the 60 week image the pectoral fins are angled downward

For effective use of I^3^S, it is important to know how frequently photographs need to be taken for accurate identification over time. To establish how increasing the time interval between databases affects the accuracy of the software's recognition scores and thus correct identification, we computed recognition scores for comparisons between images from the final database with matches in each of the preceding databases (Figure 2). For this comparison, the sample population was divided into one group of older individuals (16 weeks old at the beginning of the study) and one of younger individuals (<2 weeks old at the beginning of the study), as the rate of pattern development was suspected to vary with age. If the population had been considered as a whole, correlations between time interval and recognition score could have been confounded by age, potentially masking any relationship. Mean (±SE) recognition scores from the two groups were calculated for each database (Figure 2). The longer the time interval between databases, the greater the recognition scores, indicating that recognition degrades as the time interval between photographs increases (Spearman's rank *r*_s14_ = 0.912, *P* < 0.001; Figure 2b). No correlation was found in the older group between time interval and the recognition score (*r*_s14_ = 0.347, *P* > 0.05). Thus, correct identification of the younger, but not older, hatchlings is more difficult with increasing periods of time between photographs.

### 3.2 Genetic validation

All seven microsatellite primers amplified each locus for all of the samples tested (Table 1). Parentage analysis determined all the individuals within the source population had contributed to the photo‐recognition experimental individuals (Cat01–Cat18). As not all the individuals were full siblings, the probability of identity (pID) was calculated without the assumption of full siblingship. Both probability indices produced highly significant results, positively matching two samples, identifying them as duplicates and, therefore, the same individual. No duplicates were found within the same sampling time point, indicating an appropriate level of genetic diversity within the population for the analysis. Matching identifications were found using all seven of the microsatellite loci, with no mismatching loci.

## 4 DISCUSSION

Through the use of microsatellite markers, we show that photo‐recognition software can successfully identify individual small‐spotted catsharks during their first year of life. The use of photo‐recognition and genetic validation has been previously performed in wild white sharks, but only after the sharks were older than 1 year, when growth had stabilised (Graham & Roberts, 2007). Our study combines photographic recognition software and genetic analysis during early development and shows that photographs should be taken at intervals of 1 week for the first 8 weeks after hatching, whilst pattern development is at its most changeable. As the sharks age and pattern prominence increases, time intervals between photographs can increase to 1 month without compromising recognition score.

Despite the success of this method, certain caveats should be acknowledged. Firstly, *S. canicula* is known to change colouration based on its substrate and surroundings (Visconti *et al*., 1999). Although no such changes were observed in this study, identification in wild populations could be more difficult if the natural markings fade or change. Secondly, captive populations ensure certainty that every individual will be present in each database. It is more problematic for wild populations, where the total number of unidentified individuals is unknown. Thirdly, over long study periods, changes in body shape may pose an issue if the relative positions of the three reference points change over time. If these change, growth and scale cannot be as effectively corrected for, resulting in higher recognition scores (lower similarity). It is recommended that subsequent studies should investigate the potential effects of such changes in older small‐spotted catsharks. If recognition success decreases, increasing the frequency of the photographs will compensate. Fourthly, including multiple observers may increase recognition scores through bias in spot selection. Finally, the quality of the photograph, specifically the lighting and angle, must be consistent (Speed *et al*., 2007).

Due to the study being conducted within captivity, true relationships between individuals were easily determined by genetic identity analysis. Previously, small‐spotted catsharks have displayed levels of multiple paternity (Griffiths *et al*., 2012) and, therefore, parentage was necessary to correctly identify the differences between low variation and identity. Although the majority of individuals are closely related, either as full siblings or half siblings, no individuals had exact genotypic fingerprints or exact pattern matches.

Animal welfare is of increasing concern in captivity. Aquariums aim to reduce the number of wild‐caught individuals and become more involved in captive breeding to assist conservation efforts (Smith *et al*., 2004). Traditional identification methods such as PIT tags are less suitable at a younger ages due to the potential negative effects on animal survival, physiology or behaviour (Gibbons & Andrews, 2004). Overall this study found that small‐spotted catsharks can be identified using natural markings from hatching to 1 year of life, without invasive tagging, if photographs are taken once a week for the first 8 weeks. The I^3^S software therefore provides a free and reliable method for individual recognition where, beyond installing the software, no specialist equipment is required.

## AUTHOR CONTRIBUTIONS

H.A.S. devised the project; C.M. and S.A.H. carried out the project; B.G. and T.M. supplied the shark embryos and parentage tissue samples; N.A. and D.R. assisted with photography and tissue collection. C.M. analysed the photographic data, S.A.H. analysed the genetic data and D.R. generated the statistical analyses. All authors contributed to interpretation and writing.
